# Translational research in Uganda: linking basic science to bedside medicine in a resource limited setting

**DOI:** 10.1186/s12967-021-02747-z

**Published:** 2021-02-16

**Authors:** Richard Kwizera, Emmanuel Mande, Denis Omali, Samuel Okurut, Sheila Nabweyambo, Rose Nabatanzi, Damalie Nakanjako, David B. Meya

**Affiliations:** 1 Translational Research Laboratory, Department of Research, Infectious Diseases Institute, College of Health Sciences, Makerere University, P.O Box 22418, Kampala, Uganda; 2 Department of Medicine, School of Medicine, College of Health Sciences, Makerere University, Kampala, Uganda

**Keywords:** Translational research, Laboratory research, Translational medicine, Translational science, Bench-to-bedside research, Uganda

## Abstract

**Background:**

Translational research is a process of applying knowledge from basic biology and clinical trials to techniques and tools that address critical medical needs. Translational research is less explored in the Ugandan health system, yet, it is fundamental in enhancing human health and well-being. With the current high disease burden in Uganda, there are many opportunities for exploring, developing and utilising translational research.

**Main body:**

In this article, we described the current state, barriers and opportunities for translational research in Uganda. We noted that translational research is underutilised and hindered by limited funding, collaborations, laboratory infrastructure, trained personnel, equipment and research diversity. However, with active collaborations and funding, it is possible to set up and develop thriving translational research in Uganda. Researchers need to leverage existing international collaborations to enhance translational research capacity development.

**Conclusion:**

Expanding the integration of clinical and translational research in Uganda health care system will improve clinical care.

## Background

The definition of translational research is less clear and varies widely since it combines both aspects of basic science and clinical research [1]. However, it has been defined as a process of applying knowledge from basic biology and clinical trials to techniques and tools that address critical medical needs [2]. The term “translational research” is often used interchangeably with words such as bench-to-bedside research, translational science or translational medicine [3]. Medical advances in human health require the efficient and rapid translation of new basic scientific discoveries into effective vaccines, drugs, medical diagnostics, and new treatments options. This process in turn depends on data obtained from laboratory experiments, observational studies and public-health research that can be used to inform treatment guidelines. Translational research therefore builds on basic scientific research to create new medical interventions that can enhance human health and well-being.

Basic biomedical research often focuses on studying diseases in vitro or in vivo commonly using animal models or cell cultures in controlled laboratory experiments/environments. The word “translational” refers to the carry-over of these basic biomedical research findings from a laboratory setting into potential vaccines, diagnostics, and treatments for various medical conditions [4]. Therefore, a well-established laboratory is a key component of translational research. The idea of translational research was originally devised because of the elongated periods often taken to bring novel clinical and diagnostic discoveries into clinical practice in most health systems. Translational research strongly requires collaboration, funding, technical skills, facilities and resources that are often not readily available in a basic clinical diagnostic laboratory setting. It is for this reason that most translational research is carried out in dedicated research centres and isolated University science departments.

Translational research is fundamental in improving and advancing clinical care in regions with heavy disease burden, such as Sub-Saharan Africa and Asia. Globally, translational research is hindered by numerous factors including; basic structural and cultural impediments to innovation and collaboration, shortage of trained investigators and funding [5]. The barriers to translational research are more pressing in Sub-Saharan African countries like Uganda, hence an urgent need to build local capacity in these areas. In this article, we discuss the state of translational research, factors hindering its development and opportunities in Uganda.

## Main text

### State of translational research in Uganda

Traditionally, national treatment guidelines published by the Ugandan Ministry of Health are based on international guidelines, especially from the World Health Organisation (WHO) [6]. This has limitations of incorporating local research findings into our guidelines, especially given that WHO guidelines may have been adopted from research conducted in geographical locations with different ethnic populations, medical and cultural practices. This therefore calls for establishment of local research capacity to address local disease burdens like human immunodeficiency virus (HIV). In Uganda, the current prevalence of HIV among adults (15–49 years) is 5.7% with 23,000 AIDS-related deaths annually [7]. This has led to the establishment of robust medical research institutions with the support of a number of global funding agencies to accelerate both observational and clinical studies to improve diagnosis and treatment outcomes of patients living with HIV and other related infectious diseases in Uganda.

Uganda is one of the very few countries that have contributed most to research translation in Sub-Saharan Africa. There are many ongoing clinical studies in Uganda; some of which have informed better local and international treatment guidelines for various diseases. For example, the The Cryptococcal Optimal ART Timing (COAT) trial (ClinicalTrials.gov number, NCT01075152.) conducted in Uganda [8], whose results led to the current World Health Organization (WHO) recommendation that ART-naïve patients with cryptococcal meningitis should initiate ART 4–6 weeks following diagnosis and treatment of cryptococcal meningitis [9]. Accompanying this clinical trial were immunology studies evaluating the possible explanation for the differential mortality associated with antiretroviral therapy (ART) timing [10–12]. Such clinical trials currently provide a platform for improving outcomes for HIV-infected individuals in Uganda [13]. Originally, such clinical studies were carried out in Uganda but patients’ samples shipped to collaborators in the USA and European countries for testing, since there was lack of local capacity for translational research. However, the published findings from these clinical studies benefited both Ugandans and everyone worldwide.

Translational research is a relatively new area that has not been fully explored in the Ugandan healthcare system to improve patients’ management and treatment outcomes. There are a few translational research laboratories in Uganda most of which are localized at research centres in the central part of the country mainly carrying out research in HIV, HIV-related opportunistic infections and other infectious diseases. Some of these research centres include the Infectious Diseases Institute, Joint Clinical Research Centre, Uganda Virus Research Institute, Makerere University Walter Reed Project, Rakai Health Sciences Program, Epicentre Uganda, Uganda Cancer Institute -Fred Hutch Cancer Centre, Makerere Biomedical Research Centre of Excellence Laboratories, and MildMay Uganda. However, findings published from translational research done in these selected institutions in our setting is of high quality and conforms to recommendations cited in the developed world [8, 14, 15].

Besides HIV, other leading causes of morbidity and mortality in Uganda include Malaria, TB, and respiratory diseases, diarrhoeal, and vaccine-preventable diseases [16]. Uganda is also faced with an increasing incidence rate of mental disorders and non-communicable diseases (NCDs) including cancers, cardiovascular diseases and metabolic disorders. Maternal and perinatal conditions also contribute to the high mortality. Neglected tropical diseases (NTD’s) such as lymphatic filariasis, leprosy, schistosomiasis, soil-transmitted helminths and sleeping sickness remain a big problem in the country affecting mainly rural poor communities [16]. The burden of fungal infections is also high with an estimated 2.5 million fungal infections per year in Uganda [17], yet the index of clinical suspicion remains low [18, 19]. The growing burden of NCDs could be attributed to change in life style and the environment that necessitates research investigations tailored to the local burden. Similarly, Uganda is among the African countries with a high burden of maternal and new-born morbidity and mortality, with the current maternal mortality ratio estimated at 336 deaths/100,000 live births [20]. This has been attributed to Biological factors as well as gaps in the health care system that can be investigated through translational research. In addition, there are very few disease surveillance programs in Uganda supported by translational research, making it hard to effectively and timely inform national health policies and guidelines.

### Factors hindering the development of translational research in Uganda

In this section, we discuss the factors hindering the development of translational research in Uganda, highlighting the issues, limitations and proposed solutions (Table 1). Limited funding is a major factor that hinders development of Translational research in Uganda. Most research funding in Uganda is from external sources with limited support from the government. Many of the funders come in with already fixed ideas that do not tackle the local problems. Therefore, translational research studies that could impact local health problems rather than more global problems attract less interest from international funders. This is more common for the NTD’s and NCD’s.

**Table 1:** Summary of issues, limitations and proposed solutions

Issues	Limitations	Proposed solutions
Treatment guidelines published by the Ugandan Ministry of Health are based on international guidelines, especially from WHO	WHO guidelines may have been adopted from research conducted in geographical locations with different ethnic populations, medical and cultural practices	Establishment of local research capacity to address local disease burdens
Limited funding; Most research funding in Uganda is from external sources	Many of the funders come in with already fixed ideas that do not tackle the local problems. Therefore, translational research studies that could impact local health problems rather than more global problems attract less interest from international funders	Advocate for more research funding from Uganda government
There are very few active long-standing collaborations with external partners to support the establishment of translational research locally	Most international collaborators need well established research facilities with a good track record of research in their area of interest to extend their collaboration	Encourage local research in popular areas in order to attract collaborators. Need to develop internationally recognised research institutions and centres of excellence
Poor laboratory infrastructure	Most of the translational research laboratories at research centres are accommodated in limited spaces, most of which were not originally meant for laboratories. Such spaces cannot allow the proper infrastructure or expansion required for a basic translational research laboratory	Need to establish dedicated translational research laboratories at selected research institutions and hospitals where patients are managed
Limited trained personnel/scientists with specialized skills	Most laboratory staff are not well trained to perform the sophisticated laboratory technics	Need specialised postgraduate training in the specific areas of biomedical research
Expensive laboratory equipment	Very few laboratories can afford the up to date equipment needed to perform the sophisticated procedures	Need more funding from both local and external partners to acquire these equipment
Limited research diversity	Most laboratories only performing HIV related research	Raise awareness of the local disease burden and interest researchers to venture into other common diseases. Researchers and funding agencies should also engage private universities to get involved in translational research

Besides, there are very few active long-standing collaborations with external partners to support the establishment of translational research locally. For example, the Infectious Diseases Institute has five long-standing collaborations with the National Institutes of Health (NIH), University of California San Francisco (UCSF), University of Zurich, University of Minnesota and John Hopkins University aimed at building local capacity for translational research in Uganda. Such collaborations are essential in sustainability of translational research especially due to continued knowledge sharing through funded research or student projects between the collaborating institutions.

Another pressing challenge is poor laboratory infrastructure. Most of the translational research laboratories at research centres are accommodated in limited spaces, most of which were not originally meant for laboratories. Such spaces cannot allow the proper infrastructure or expansion required for a basic translational research laboratory.

Limited trained personnel/scientists with specialized skills to perform the sophisticated laboratory technics in immunology, microbiology, molecular biology and pharmacology, likewise hinders the progress of translational research in Uganda. It is more critical to have well-trained scientists with the ability to conduct research and troubleshoot assays rather than just having laboratory technicians to operate the equipment.

The translational research laboratories need expensive and up to date equipment for the smooth operations. Even with the few translational research laboratories in place, there is still limited research diversity with most laboratories only performing HIV related research. This limits the scope and interest of trainings available. Yet, communicable diseases, which account for over 50% of morbidity and mortality, dominate Uganda’s burden of disease.

### Opportunities for exploring, developing and utilising translational research in Uganda

Uganda has 155 hospitals (private and public) and of these, two are national referral hospitals, 117 are Regional referral hospitals and 139 are General Hospitals. These hospitals are distributed in 134 districts in all regions of the country with the potential for creating collaborations between research scientists and these hospitals for more representative population and findings for the whole country. Researchers can leverage current surveillance programs and databases in these hospitals to build translational research capacity in the country.

The existing HIV-based translational research laboratories have most of the basic facilities and equipment that are needed to investigate other infections of interest to the local disease burden. This can encourage research diversity. Similarly, these existing HIV-based facilities can be used as benchmarks and training facilities by government for setting up other translational research laboratories at various hospitals. Besides, a well-established translational laboratory with highly specialised training among the individuals can enhance local research capacity. This is the most critical missing link in Uganda. This in turn creates global research leaders capable of doing independent research at an international level. We are in an era of evidence-based medicine and translational research is a key component.

Embracing translational research would potentially lead to specialisation in different areas resulting in research diversity and high-quality output. This in turn helps to inform both local and international treatment guidelines based on data from various research studies. This can also lead to local innovations in diagnostics, vaccines and other pharmaceuticals. Similarly, the high-quality data can also inform international guidelines. Additionally, high quality translational research output can lead to development of internationally recognised research institutions and centres of excellence, which in turn accelerate the transition of basic research findings into clinical practice. Translational research is a vital component of active surveillance for emerging diseases to monitor and detect disease outbreaks. Translational research encourages international collaborations and attract research funding. Translational research laboratories help to validate new medical devices and diagnostics before rolling out their use in the general population.

### Overview of the Translational Research Laboratory at the Infectious Diseases Institute

The Translational Research Laboratory of the Infectious Diseases Institute at the College of Health Sciences, Makerere University, has been functional since June 2009. The laboratory contributes to both translational and clinical research studies and supports trainees mostly at Makerere University (including post-doctoral and PhD fellows, Masters’ students and undergraduates). The services offered by the laboratory include biorepository, point of care testing, clinical diagnostics and research in microbiology, immunology, molecular biology, pharmacokinetics and pharmacogenomics. The laboratory is equipped with state-of-the-art machines like Multiplex Polymerase chain reaction (PCR) platforms, High performance liquid chromatography machines coupled with UV detection (HPLC-UV), LCQ Fleet mass spectrometer, BD FACSCanto™ II, Luminex MAGPIX^®^ system, BD BACTEC™ 9050 blood culture system and DNA extraction and quantification instruments, point of care molecular equipments like the Cepheid GeneXpert and Biofire FilmArray multiplex PCR system. The laboratory staff have a variety of expertise and post-graduate training in Mycology, Immunology, Microbiology, Molecular biology, Pharmacology and Bioinformatics, supporting a number of research studies including the national surveillance program on Gonorrhoea [21]. From 2013 to 2020 the laboratory collaborated with internationally renowned universities and organisations such as University of Minnesota, John Hopkins University, University of California San Francisco, University of Zurich and the National Institutes of Health (NIH), in support of numerous research projects. All of these projects have the potential to lead to better treatment outcomes for patients in Uganda and globally. Some of the key outputs for this laboratory are; Validation of PIMA^®^ CD4 point-of- care machine which encouraged countrywide roll out [22]; Validation of the IMMY CrAg^®^ LFA which led to its FDA approval and WHO recommendation for its use [23–26]; PK interaction finding between rifampicin and artemether which led to product label change for Coartem [27].

## Conclusions

Translational research remains largely unexplored in Uganda, yet it is fundamental in enhancing human health and well-being. Given the existing high disease burden in Uganda, there are numerous opportunities for translational research despite various barriers. Current limitations in funding, research collaboration, laboratory infrastructure, trained personnel, equipment, and research diversity pose significant challenges to the growth of translational research in Uganda. Researchers need to leverage active international collaborations and new technologies to enhance the capacity of translational research. We believe that building translational research capacity will lead to improvement in clinical care, which will translate into benefits for the healthcare system. These benefits will accrue from increased government funding, which could support the establishment of translational research laboratories at selected research institutions and hospitals where patients are managed. Researchers and funding agencies should also engage private universities to get involved in translational research.

## Data Availability

The authors confirm that all data underlying the findings are fully available without restriction and can be availed by contacting Mr. Richard Kwizera (kwizerarichard@ymail.com).

## Abbreviations

WHO: World Health Organisation
HIV: Human immunodeficiency virus
AIDS: Acquired immunodeficiency syndrome,
COAT: Cryptococcal Optimal ART Timing
ART: Antiretroviral therapy
USA: United States of America
TB: Tuberculosis
NCD: Non-communicable diseases
NTD: Neglected tropical diseases
NIH: National Institutes of Health
UCSF: University of California San Francisco
PCR: Polymerase chain reaction
HPLC–UV: High performance liquid chromatography machines coupled with UV detection
